# Surgical treatment of nonuremic calciphylaxis: a case report and review of literature

**DOI:** 10.1080/23320885.2022.2145962

**Published:** 2022-12-15

**Authors:** Minami Tamagake, Munetomo Nagao, Chieko Miura, Yoshimichi Imai

**Affiliations:** Department of Plastic and Reconstructive Surgery, School of Medicine, Tohoku University, Sendai, Miyagi, Japan

**Keywords:** Nonuremic calciphylaxis, surgical treatment, debridement, skin grafting

## Abstract

Calciphylaxis is characterized by extremely painful skin ulcers and develops in patients with or without severe kidney diseases. Herein, a female patient without renal dysfunction developed calciphylaxis in the right lower extremity and underwent successful surgical debridement and split-thickness skin grafting. Nevertheless, prompt wound evaluation and proper surgical approaches are essential.

## Introduction

Calciphylaxis is a rare and extremely painful disease characterized by painful skin ulcerations and necrosis in various areas of the body and has a high mortality rate, with 1-year mortality rate reported as 45–80% [1]. Calciphylaxis is typically diagnosed in patients with end-stage kidney disease, affecting up to 4% of long-term dialysis patients, also known as calcific uremic arteriolopathy (CUA) [1]. Exceptionally, some nonuremic cases of calciphylaxis have also been reported. Although wounds of calciphylaxis are often difficult to heal, effective treatment has not yet been established. Calcification of the medial arterioles in subcutaneous and adipose tissues leads to vascular thromboses, resulting in ischemic necrosis or ulcerations [2]. Despite the high mortality rate, mainly due to sepsis attributed to wound infections [3], aggressive surgical treatment, such as extensive debridement or reconstructive surgery, is uncommon because ischemic tissue is thought to delay healing. On the other hand, leaving infected calciphylaxis lesions can lead to sepsis; therefore, infected or necrotic tissue may require emergent surgical debridement. We report the treatment over 2 years of a patient with nonuremic calciphylaxis caused by trauma, who was successfully treated with aggressive debridement and reconstruction using split-thickness skin grafting.

## Case report

**Figure 1.**
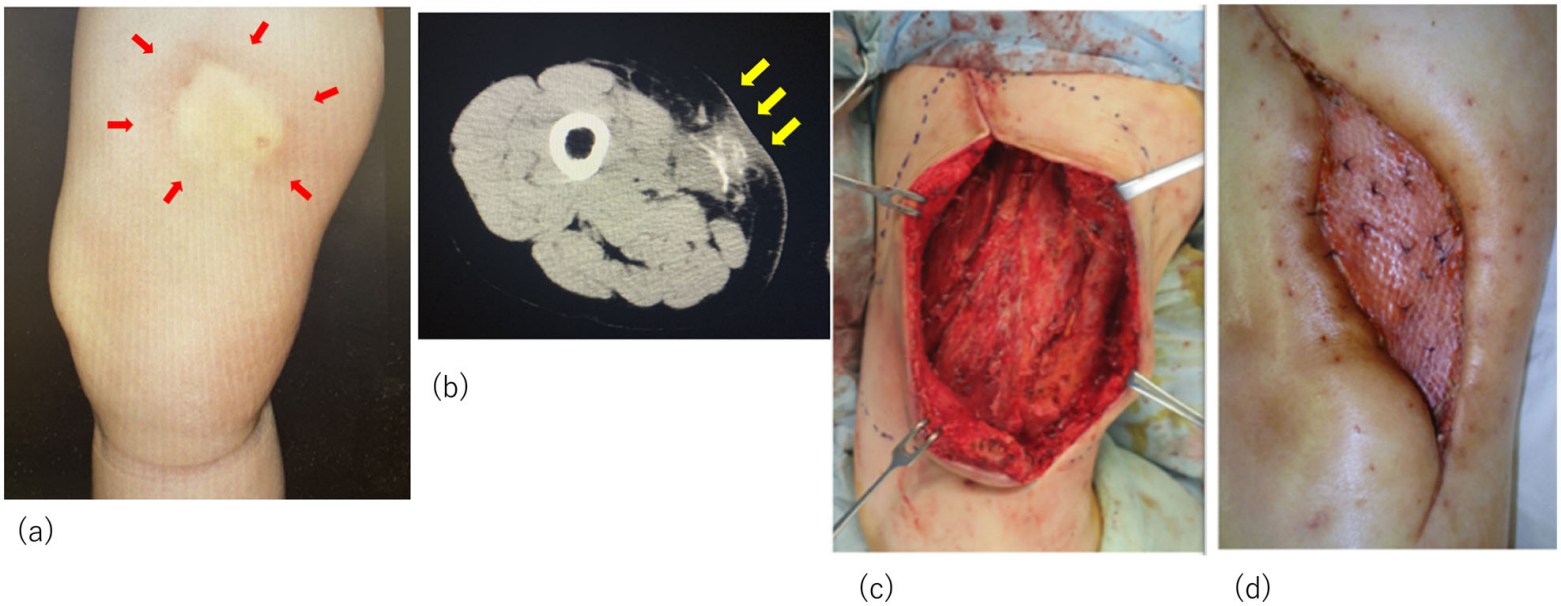
Initial examination and surgery of the patient. (a) The initial lesion of the thigh; arrows indicate the indurated nodule. (b) Computed tomography images indicating calcification and inflammation. Arrows indicate the calcification. (c) Intraoperative view of the thigh after radical debridement. (d) Successful placement of the skin graft.

We present the case of a 55-year-old female patient with a history of breast cancer, adenomyosis of the uterus, and gastroesophageal reflux, with normal renal function, and not taking warfarin. She initially bruised the lateral area of her right thigh, which became a painful and indurated nodule with redness (Figure 1a). Gradually, she experienced severe radiating pain and numbness over her entire right lower limb and was referred to a local orthopedic hospital. The relevant medication history included agents containing activated vitamin D3 and calcium supplementation for osteoporosis. Computed tomography (CT) revealed extensive soft tissue calcification and inflammation reaching the sartorius muscle at the initial lesion site (Figure 1b). A partial excision biopsy was performed, and pathological examination revealed diffuse necrosis and calcareous deposits in the lesion.

The biopsy wound developed ulceration, leading to further pain, inflammation, intermittent pustulous exudates, and a complete lack of progression towards healing. In addition to antibiotic medication and analgesic therapy, removal of necrotic tissue from the initial lesion was necessary to facilitate wound healing, and the patient was referred to the Department of Plastic Surgery for more intensive wound care.

Laboratory data showed no abnormalities in serum calcium, phosphorus, or parathyroid hormone levels. Additionally, there were no obvious abnormal findings in the examination results of the coagulation system, anti-nuclear antibodies, anti-DNA antibodies, complements, tumor makers, antibodies, or antigens of some infectious diseases (Table 1). Cultures of the necrotic tissue contained bacteria commonly found on the skin; there was no indication of systemic fungal infections, nontuberculous mycobacterial infection, or necrotizing fasciitis. Systemic CT angiography, positron emission tomography –CT, and inspection of skin perfusion pressure were performed to rule out ischemic diseases and malignant tumors.

**Table 1.** Laboratory date at the time of admission High test values are indicated in red.

WBC	4.8 × 10³/μL
Neutro	56.0%
Lymph	36.0%
Mono	4.0%
Eosino	4.0%
Baso	0.0%
※ No heterozygous lymphocyte	
RBC	3.58 × 10^6^/μL
PLT	16.2 × 10^4^/μL
Hb	11.1 g/dL
Alb	3.5 g/dL
Cre	0.37 mg/dL
UN	8 mg/dL
eGFR	134
γ-GTP	18 U/L
ALT	22 U/L
AST	18 U/L
ALP	156U/L
HbA1c	5.6%
CRP	0.78 ng/dL
Na	142 mmol/L
K	3.8 mmol/L
Ca	9.5 mg/dL
P	3.8 mg/dL
PTH	26.6 pg/mL
PTHrP	1.1 pmol/L
Renin activity	0.8 ng/mL/hr
Aldosterone	15.3 ng/mL
Calcitonin	0.5 pg/mL
1,25 dihydroxy vitamin D3	58.0 pg/dL
Coagulation System:	
PT	1.01 INR
APTT	28.9 sec
ATIII	86%
D-Dimer	1.6 μg/dL
Anti-nuclear antibodies:	
Anti-RNP	(–)
Anti-Sm	(–)
Anti-SSA	(–)
Anti-SSB	(–)
Anti-Scl 70	(–)
Anti-Jo-1	(–)
anti-DNA antibodies:	
Anti ds-DNA	(–)
Anti ss-DNA	(–)
Complements:	
C3	130 mg/dL
C4	33.8 mg/dL
CH50	59.3 U/mL
Tumor makers:	
DUPAN-2	5 U/mL
Span-1 antigen	10.4U/mL
CEA	0.7 ng/mL
CA19-9	6.5 U/mL
Infectious Disease:	
HIV-1,2 antibody	(–)
HBs antigen	(–)
HBs antibody	(–)
HCV antibody	(–)
Treponema antibody	(–)
Other:	
Rheumatoid factor	6.3 IU/mL
Cryogloblin undetectable	
IgG	1197 mg/dL
IgA	155 mg/dL
IgM	122mg/dL
IL-2	229 U/mL
Indirect Coomes test	(–)
Direct Coomes test	(–)

The patient was asked to discontinue the medication of vitamin D and calcium.

Despite the lack of a definitive diagnosis, surgical debridement of the infectious lesion, necrotic adipose tissue, and part of the degenerated sartorius muscle was necessary to prevent sepsis (Figure 1c). Subsequently, negative wound pressure therapy with instillation and dwell time (NPWTi-d) was initiated. Four weeks of NPWTi-d therapy induced healthy granulation bed formation in preparation for reconstructive procedures. The wound was covered with a meshed split-thickness skin graft at a ratio of 3:1 and secured with a tie-over dressing. The donor site was the lateral area of the left thigh. The skin graft was successful, and the patient was discharged after a month of rehabilitation and wound treatment with ointments (Figure 1d).

**Figure 2.**
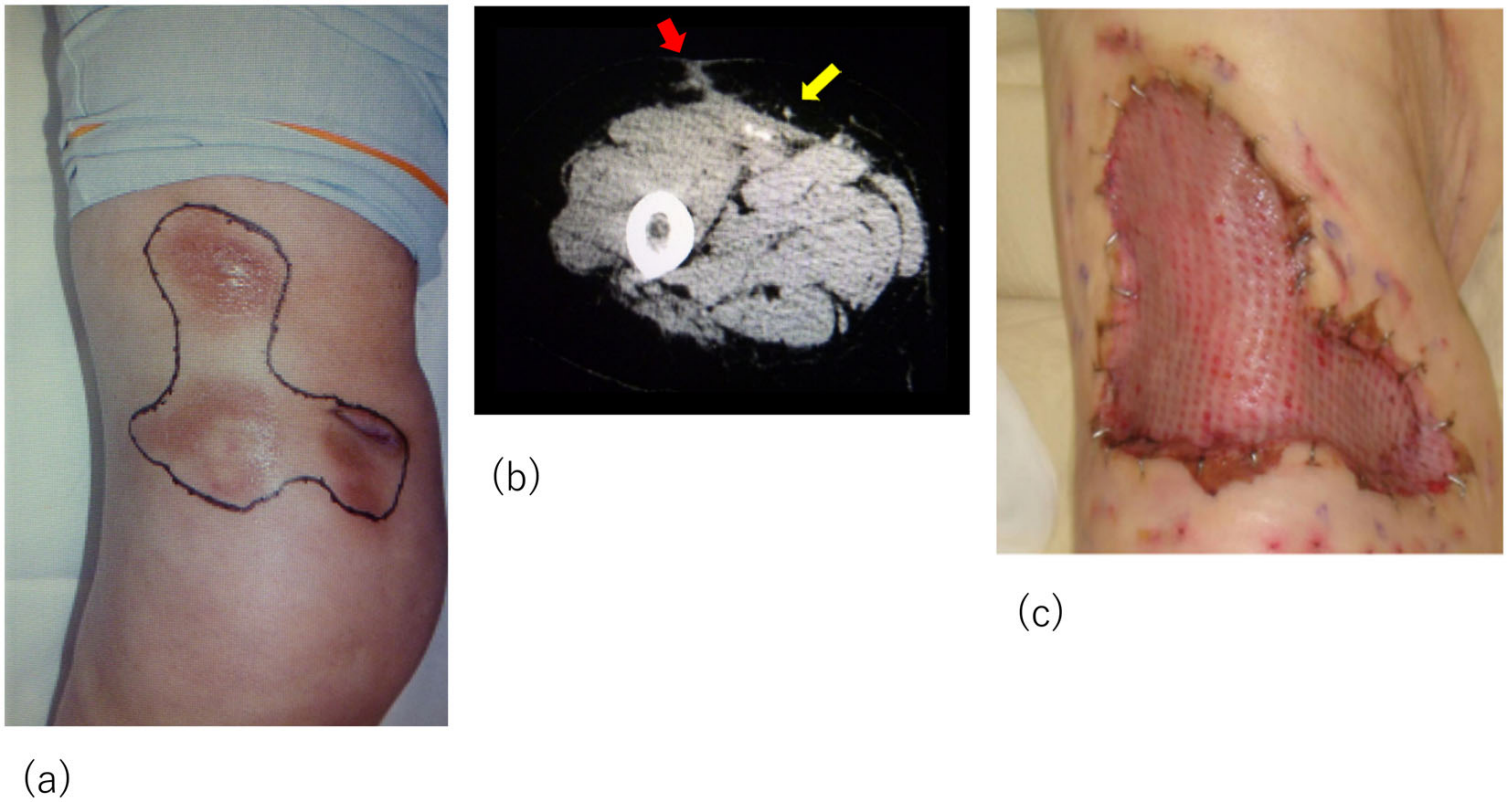
Recurrent lesion in the middle of the right thigh. (a) The lesion in the middle of the right thigh; obvious nodules with redness are marked. (b) Computed tomography images of the lesion: yellow arrow indicates the calcification in the muscle and red arrow indicates inflammation spreading towards subcutaneous tissue. (c) Meshed split-thickness skin graft taken successfully.

**Figure 3.**
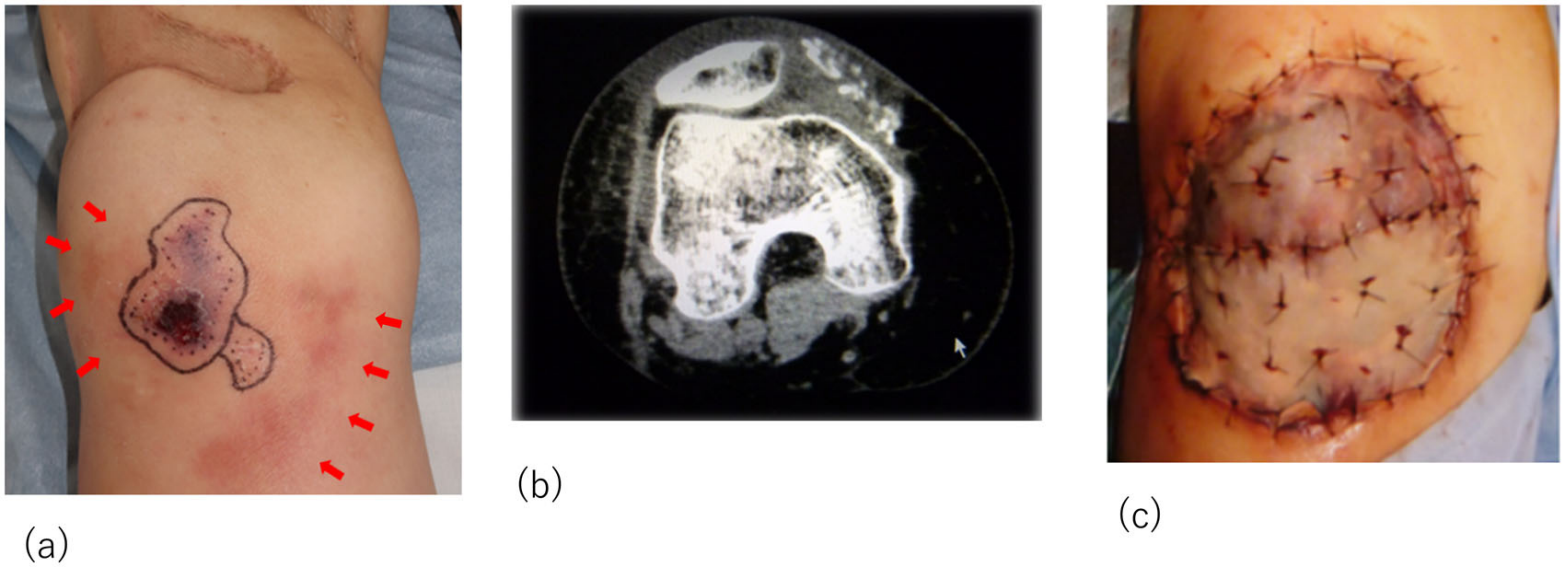
Recurrent lesion on the right knee. (a) The lesion on the right knee: obvious nodule with purpura and skin necrosis is marked. Arrows indicate the inflammation spreading around the knee. (b) Computed tomography images showing calcification and inflammation at the knee joint. (c) The skin graft was applied as a sheet in the knee joint area.

**Figure 4.**
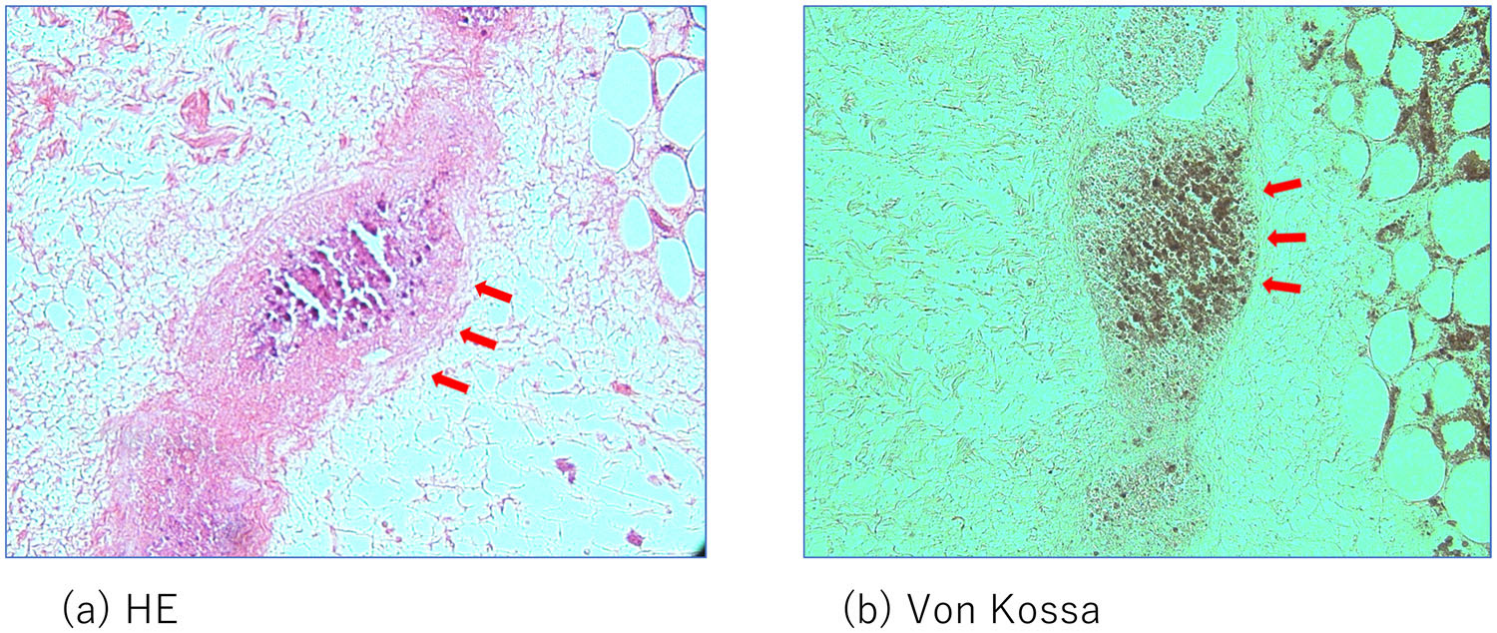
Pathological examination: arrows indicate the calcification in the small vessels in the subcutaneous tissue.

**Figure 5.**
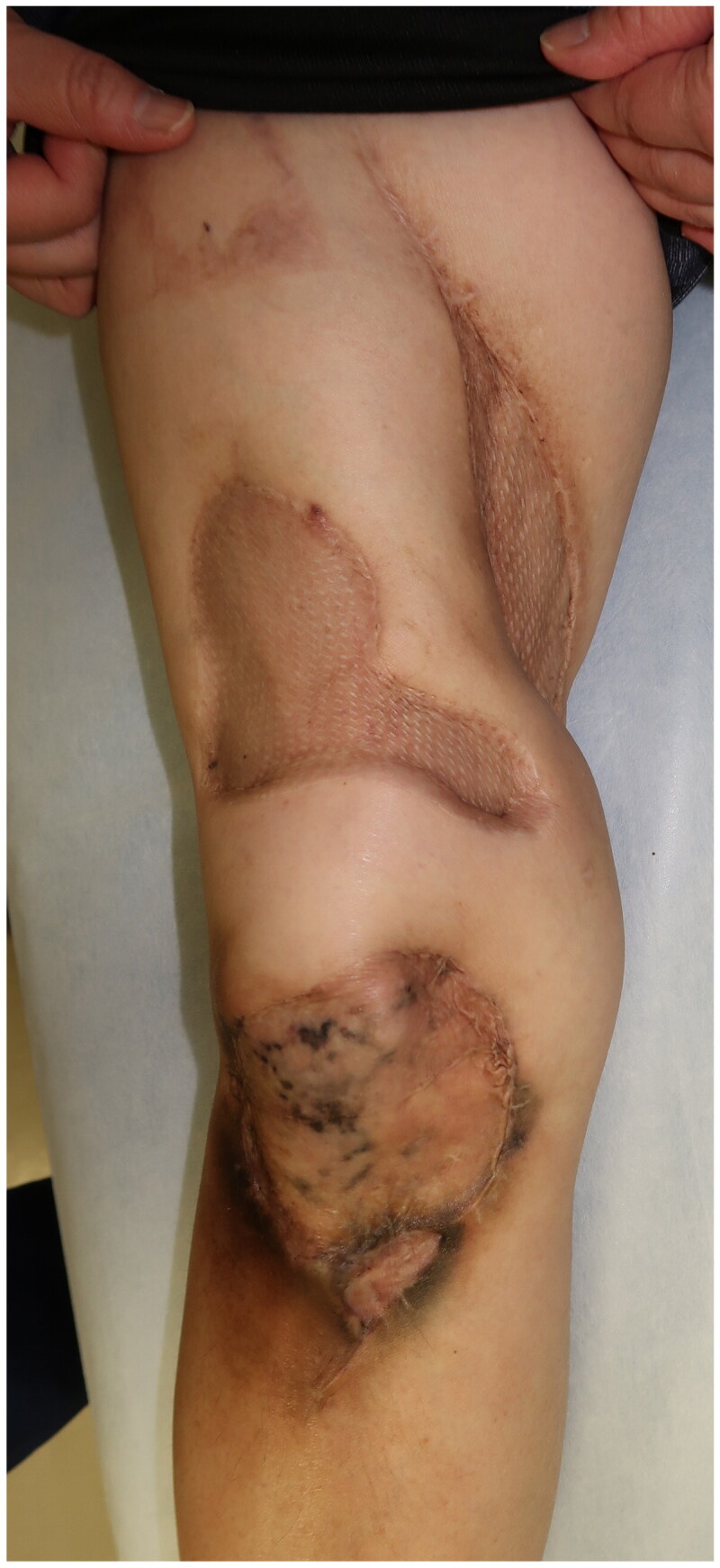
Six months after the last surgical treatment, the patient had no apparent wound infections or new ulcerations.

Although the initial treatment was successful, other lesions occurred in the anterior part of the right thigh and right knee. The patient required surgical debridement and skin grafting several times over the course of approximately 2 years (Figures 2a,b and 3a,b). All wounds progressed in the same manner as the previous lesion, with painful induration, refractory ulceration, and purulent exudate, indicating necrosis of the deep soft tissue. Surgical debridement was performed to remove the necrotic area, and NPWTi-d therapy was initiated in preparation for skin grafting. After achieving good granulation, meshed split-thickness skin grafting was performed on the anterior part of the right thigh (Figure 2c), and the skin graft was applied as a sheet in the knee joint area (Figure 3c). The grafts of both areas successfully took . The pathological specimen obtained from the third debridement revealed calcification and microthrombosis of the small dermal and subcutaneous arteries and arterioles. The calcification likely caused ischemic necrosis of the subcutaneous tissue, a characteristic finding in calciphylaxis (Figure 4). After all the wounds healed, intravenous sodium thiosulfate infusion was occasionally administered to prevent recurrence in the outpatient clinic whenever signs of inflammation appeared on the scar. Since the last surgical treatment, the patient has not had any obvious wound infection or new ulceration for 6 months (Figure 5).

## Discussion

Nigwekar et al. [4] conducted a systematic review of the literature on case reports and case series of nonuremic calciphylaxis. Among the 36 patients included, primary hyperparathyroidism (27.8%), malignancy (22.2%), alcoholic liver disease (16.7%), and connective tissue disease (11.1%) were the most frequent causes of calciphylaxis in patients without renal dysfunction. Moreover, factors associated with calciphylaxis, including corticosteroids (61.1%), warfarin (25.0%), diabetes (22.2%), albumin or blood transfusion (19.4%), and protein C or S deficiency (11.1%), have been reported. As seen in our patient, trauma leading to cutaneous lesions was reported in only two cases. In some patients, laboratory data showed abnormalities in serum calcium, phosphorus, and parathyroid hormone levels, although the majority of patients had normal levels.

Our patient did not have any of the aforementioned factors, and her laboratory data did not show any abnormalities in serum calcium, phosphorus, or parathyroid hormone metabolism. A possible cause of calciphylaxis in this case was the sustained use of vitamin-D receptor activators and calcium supplementation for osteoporosis. In CUA, correlation has been established between these medications and the development of calciphylaxis [5,6]. In addition, Storan et al. reported a similar nonuremic case with no typical risk factors triggered by calcium and vitamin D supplementation [7].

In nonuremic calciphylaxis, treating the underlying etiology and removing or discontinuing potential predisposing factors should be the first-line treatment. Parathyroidectomy in patients with hyperparathyroidism, strict control of blood glucose levels in patients with diabetes mellitus, and discontinuation of corticosteroids and warfarin were effective. Correcting abnormalities in calcium, phosphorus, and parathyroid hormone metabolism is also beneficial in patients with abnormal laboratory parameters. Wound treatment with surgical and chemical debridement, empiric antibiotic therapy, pain control, hyperbaric oxygen therapy, and sodium thiosulfate infusion therapy were also effective for nonuremic calciphylaxis.

Aggressive surgical wound debridement is often controversial in the treatment of calciphylaxis [8]. Some surgical procedures for investigation or treatment, such as incisional biopsy, incisional drainage, and even insulin injection, were reported to induce calciphylaxis [4]. Patients with calciphylaxis have poor healing potential due to ischemia and comorbidities, such as diabetes mellitus or obesity. However, considering that the primary cause of mortality is sepsis caused by an infected wound, aggressive wound care to prevent infection of necrotic tissue or eschar is essential. Removal of necrotic tissue by surgical or chemical debridement has been recommended in several studies [9].

Nigwekar et al. recommended surgical debridement when infected necrotic tissue remains in calciphylaxis wounds [10]. Large necrotic areas with a high infectious risk should also be considered for surgical debridement rather than conservative treatment. On the other hand, non-infected wounds with stable and dry eschar are better managed with chemical debridement than with surgical debridement. As in our patient, deep ulcer shaving combined with NPWT and split-thickness skin transplantation has been described for calciphylaxis wounds on the extremities. Wollina et al. reported that aggressive debridement and closure with split-thickness skin grafting could not only prevent early deep skin infection but also reduce pain in calciphylaxis wounds on lower extremities [11].

Several cases of surgical debridement combined with reconstructive procedures have been reported (Table 2). All of the cases survived and gained wound healing and rehabilitation despite several comorbidities, indicating that surgical approaches are valuable for survival in patients with calciphylaxis. In most cases, the operators chose skin grafting as the reconstructive technique. We also performed reconstructive surgery by skin grafting with good results, while considering local or free flaps as reconstructive options. However, since calciphylaxis can be triggered by trauma, there was concern that new lesions might develop at the donor site. In addition, the recurrence of calciphylaxis lesions at the recipient site may make it difficult to achieve skin graft engraftment. Even if reconstructive surgery with local or free flap was successful, it was thought that the thicker tissue would cover the wound, making early detection of recurrent calcification difficult.

**Table 2.** The list of previous case reports.

Case report	Age/Sex	Localization	Comorbidity	Surgical treatment	Wound	Other treatment
Solanky et al. [12]	42/F	Lower limb	・Cirrhosis ・Post operation of vulver cancer ・Lower extermity lymphedema	After debridement, preparation of granulation by dermal matrix combined with NPWT, Reconstructed with split thickness skin grafting.	Perfect healing	－
Stanciu et al. [13]	31/M	Abdomen Thigh Buttock	・Thrombophlebitis (treated with intravenous heparin) ・Malnutrition ・Obesity ※transient episode of acute renal failure	After debridement, associating healing with NPWT, Reconstructed with skin graft.	Perfect healing	・STS ・Nutritional treatment
Tsolakidis et al. [14]	21/M	Abdomen Thigh Buttock	・Pulmonary embolism (Phenprocoumon use) ・Obesity ・Diabetes mellitis ・Hypothyroidism ・ATIII dyficiency ・DCM	After debridement, tried to reconstruct with local flap and skin graft. Flaps and grafts were unsuccessful, but buried skin grafting led the wound healing.	Healing with scar	・STS ・Pamidronato
Bhamidipati et al. [15]	38/F	Abdomen Thigh Buttock Right flank	・Post obesity surgery ・Malnutrition	Aftrer debridement, reconstructed with Amnion-Chorion Stem Cell Grafting and NPWT.	Healing with scar	・Nutritional treatment

This led us to choose skin grafting as a reconstructive technique, which requires less donor sacrifice and facilitates the observation of recurrence findings.

Weening et al. conducted a retrospective study of 64 patients with calciphylaxis regarding survival rates in several therapeutic strategies. In that report, the 1-year survival was 61.6% for patients treated with surgical debridement, in contrast with 27.4% for patients treated with conservative wound care (*p*＝0.008) [16].

The patient reported by Stanciu et al. Table 2 was treated at the burn unit to access multidisciplinary therapy. Dado et al. indicated the great benefit of treating patients with calciphylaxis at the burn center or intensive care unit because they often require a multi-faceted approach, such as intensive wound care, surgical approach, treatment of sepsis, nutritional therapy, correction of abnormalities in laboratory parameters, and anticoagulant therapy, similar to burn injury or necrotizing fasciitis [17].

Nevertheless, early evaluation and proper treatment of wounds by surgeons highly experienced in managing complex wounds are essential in the treatment of calciphylaxis.

## Conclusions

We presented the case of a patient with nonuremic calciphylaxis, who was successfully treated with aggressive debridement and split-thickness skin grafting. This case suggests that prompt removal of necrotic or infected tissue and reconstructive surgery could prevent systemic infections.
